# Personalizing treatment of pancreatitis-associated chronic pain: the need for an integrated omics approach

**DOI:** 10.1007/s00011-026-02219-4

**Published:** 2026-04-17

**Authors:** Cole Myers, Cheyenna M. Espinoza, Aaron Clarke, Elizabeth R. Lusczek, Feng Xie, Brian T. Steffen, Zeribe C. Nwosu, Guru Trikudanathan, Geetha Saarunya, Melena Bellin, Gregory Beilman

**Affiliations:** 1 Department of Surgery, University of Minnesota, 420 Delaware Street SE, Minneapolis, MN 55455 USA; 2 Department of Pediatrics, University of Minnesota, Minneapolis, MN USA; 3 Department of Medicine, University of Minnesota, Minneapolis, MN USA; 4 Department of Biomedical Sciences, University of Minnesota, Duluth, MN USA; 5 Department of Molecular Biology and Genetics, Cornell University, Ithaca, NY USA

**Keywords:** CP, Pain phenotypes, Multi-omics, Proteomics, Metabolomics, Machine learning, Biomarkers, Precision analgesia

## Abstract

**Background:**

Chronic pancreatitis (CP) is a progressive inflammatory disorder characterized by debilitating chronic pain and substantial healthcare burden. Pain mechanisms in CP are heterogeneous and incompletely integrated into clinical decision-making. This narrative review synthesizes data from human CP cohorts and complementary experimental models to summarize inflammatory, neuropathic, and metabolic drivers of pancreatitis-associated pain and to evaluate how integrated multi-omics approaches may enable mechanism-based precision management. Current treatment relies on lifestyle modification, anatomy-guided interventions, stepwise pharmacologic escalation, and surgery for refractory cases. Emerging ion-channel–targeted therapies show promise, but inconsistent patient selection and limited biomarker guidance constrain therapeutic precision.

**Findings:**

CP-associated pain arises from convergent inflammatory, neuroimmune, neuropathic, and metabolic pathways that promote peripheral and central sensitization with sustained neuroplastic remodeling. Advances in clinical phenotyping have improved characterization of pain subtypes; however, integration of biologic data remains limited. Genetic association studies increasingly implicate pathways linked to severe or persistent pain phenotypes. Omics investigations have identified candidate genomic, proteomic, and metabolomic signals that may support biologically informed stratification and treatment prediction. Nevertheless, most studies are cross-sectional, modality-specific, and derived from heterogeneous cohorts with inconsistent endpoints and limited external validation.

**Conclusions:**

Integration of rigorous clinical phenotyping with longitudinal, multi-omics modeling provides a framework for developing testable, mechanism-based biomarkers to guide personalized analgesic and procedural strategies while supporting opioid-sparing care. Priorities include harmonization of multicenter datasets, standardized and longitudinal pain outcome measurement, expanded paired biospecimen collection, and external validation of predictive models. Such efforts may enable biologically grounded pain stratification and facilitate translation of biomarker-guided decision tools into routine clinical practice.

## Introduction

Chronic pancreatitis (CP) is an uncommon but high-burden disease with an annual incidence ranging from 5–12 cases per 100,000 person-years, and a global incidence of approximately 10 per 100,000 person-years [1–4]. Pain is a predominant feature of CP and is experienced by 80–90% of patients [2, 3, 5]. Inadequately treated pain can be debilitating, with detrimental effects on financial and social well-being, underscoring the need for improved strategies to address pancreatitis—associated chronic pain [6–9]. Management of CP-associated pain begins with lifestyle interventions to reduce disease exacerbations or progression and consideration of endoscopic interventions when anatomic duct obstruction by stones or strictures is present [7]. Refractory pain is managed using a stepwise analgesic ladder, with therapy incrementally escalated until adequate pain relief is achieved. When medical therapy fails to achieve adequate pain control, surgical therapy is considered [7]. Although surgical therapy achieves pain relief in 68–80% of patients at long-term follow-up, approximately 12% require reoperation and outcomes are less favorable in patients with preoperative opioid use [10, 11]. Additionally, pancreatic morphology and risk factors associated with initial pancreatitis events do not reliably predict pain severity or pattern, though morphology guides selection of appropriate interventional approaches [12–14].

There has been increasing appreciation that CP patients experience a diverse degree of pain and show variable responses to current treatment modalities [5, 15, 16]. The underlying mechanisms of chronic pain are thought to be secondary to several parallel processes involving inflammation, prolonged neuropathic stimulation leading to neuronal remodeling with ensuing hyperexcitability, and neuronal damage [17, 18]. These neurologic changes are coupled with neuronal metabolic dysfunction that encompasses ATP depletion and oxidative stress, while calcium imbalance in pancreatic cells contributes to disease progression [19, 20]. Recent genomic studies highlight the roles of several underlying genetic mechanisms that further contribute to the complexity of pancreatitis-associated chronic pain [21].

While there has been advancement in the clinical assessment and phenotyping of pain in pancreatitis -associated chronic pain, there is a need to link clinical manifestations to underlying biologic mechanisms [21, 22]. Integrated omics approaches and modern computational techniques provide an avenue to address these knowledge gaps while also providing the means to predict clinical outcomes based on an individual’s underlying biology [22, 23]. In this review, we identified knowledge gaps in the literature that can be targeted using an integrated omics approach. We have also highlighted clinical gaps in the management of CP pain where an omics-based approach can lead to improved pain management. This review further explores how this approach may identify new therapeutic targets and allow for the development of models and biomarkers that predict treatment response in a heterogeneous CP population [24, 25].

## Clinical variability of CP pain

Many first-line analgesic strategies target nociceptive and inflammatory pain, which predominates early in pancreatitis [2, 3, 7]. Effective analgesia in CP requires a comprehensive understanding of the mechanisms driving the development and maintenance of chronic pain, including progressive neural remodeling and injury [2]. Experimental and human data demonstrate sensitization and hyperexcitability of pancreatic afferent pathways in CP, characterized by altered ionic currents in pancreas-innervating dorsal root ganglion neurons and enhanced neuropeptide-mediated neurogenic inflammation [2, 3, 26]. These neuroimmune interactions contribute to pancreatic neuritis and structural neural remodeling marked by immune cell infiltration of intrapancreatic nerves, increased perineural mast cells, and pain-associated chemokine signaling providing a mechanistic basis for neuropathic pain features in a subset of patients [27, 28]. Although neurophysiologic, metabolic, and genomic pathways have been investigated individually, integrative evidence defining how these processes converge to modulate pain intensity and chronicity remains limited, as does understanding of how they shape clinical pain phenotypes [21, 22]. A more precise characterization of pain phenotypes and their dominant mechanisms will help identify targeted, mechanism-based therapies for specific patient subgroups [5, 16].

Clinical manifestations of chronic pain in CP are highly heterogeneous, reflecting variability in disease duration, episode frequency and severity, and underlying etiology [2, 3]. This heterogeneity contributes to divergent pain trajectories and variable treatment responses. Despite this complexity, standardized phenotypic frameworks that integrate etiologic, biologic, and clinical parameters remain underdeveloped [7, 29–31]. Most mechanistic studies have examined neurophysiologic, metabolic, or genomic pathways in isolation, with limited integrative evidence defining how these processes interact in the context of pancreatitis-associated chronic pain or how they map onto distinct clinical pain phenotypes [19, 22, 29, 32, 33]. The absence of a unified, mechanism-informed classification system limits the development of targeted, individualized analgesic strategies.

Over the past decade, clinical and translational efforts have sought to define biologic and clinical phenotypes that capture the heterogeneity of CP [5, 15, 25]. Clinical instruments have been developed to distinguish nociceptive from neuropathic pain and to detect central sensitization, which often signals more refractory, long-term pain [5, 15]. Despite these advances, most phenotyping to date has focused on clinical manifestations that indirectly reflect biology [5]. More precise characterization will require integrating biological data such as genomic, proteomic, and metabolomic profiles to better predict pain responses to specific interventions [22].

A recent genomic study by Dunbar et al. identified candidate genes associated with heterogeneity in pancreatitis-related chronic pain and organized these findings into biologically coherent pathways that may underlie distinct clinical phenotypes [21]. The transition from nociceptive to neuropathic pain is thought to involve peripheral and central sensitization, neuroimmune and glial activation including microglia-driven signaling and downstream cytokine and brain-derived neurotrophic factor (BDNF)-mediated synaptic plasticity [34–37].

In CP, mechanistic pain research has advanced most prominently in clinical phenotyping. Pancreatic quantitative sensory testing (QST) has identified reproducible pain phenotypes and demonstrated predictive value for pregabalin response, while the Patient-Reported Outcomes Measurement Information System (PROMIS) derived nociceptive versus neuropathic pain-quality phenotypes and CP-specific instruments such as Comprehensive Pain Assessment Tool (COMPAT) and COMPAT-Short Form have further refined patient stratification [5, 38–40]. Genetic association studies have also identified variants linked to severe or constant pain patterns [21, 32]. However, integrated longitudinal multi-omics datasets that explain interpatient biological variation and robustly link it to standardized pain trajectories and treatment response remain limited [15, 22].

Developing predictive models will require rigorous phenotyping across clinical and biological data streams, followed by harmonization into high-dimensional datasets capable of capturing clinically meaningful biological variation [22, 41]. By leveraging these potential integrated predictive models, clinical management of CP pain could evolve from the traditional trial-and-error approach toward a more evidence-based, precision paradigm [4, 22]. This transition would enable early identification of patients at risk for developing central sensitization and allow for timely, targeted interventions [15]. In the following section, we explore the mechanistic domains and molecular contributors underlying CP pain including neurophysiologic, metabolic, and genomic pathways to better understand how these biologic processes intersect and inform the development of individualized, mechanism-based therapeutic strategies.

## Mechanistic domains and molecular contributors to CP pain

Achieving effective analgesia in CP requires understanding the diverse pathophysiologic mechanisms that drive the development and persistence of chronic pain, including progressive neural remodeling and injury [2]. Although neurophysiologic, metabolic, and genomic pathways have been studied individually, integrative evidence defining how these processes intersect remains limited [22]. Furthermore, insufficient knowledge exists regarding how these biologic pathways shape the clinical manifestations of chronic pain [5]. A more precise characterization of pain phenotypes and their dominant mechanisms will help delineate which therapeutic modalities are most effective for specific patient subgroups [16]. Multi-omics approaches have been explored in other chronic pain syndromes and have facilitated the development of more personalized treatment strategies [42]. Integrating high-dimensional omics datasets that capture relevant biologic processes with standardized clinical outcomes offers the potential for more biologically informed decision-making when matching therapies to distinct CP subgroups [22].

### Neurophysiology and ion channels

Several ion channels represent promising therapeutic targets in CP, as they play central roles in nociceptive signal transduction, action potential propagation, and neurotransmitter release. Together, these processes culminate in the perception of pain in response to noxious stimuli [43–45]. Multiple nociceptor ion channels expressed by pancreatic sensory afferents are implicated in CP pain, contributing to visceral hypersensitivity and pain behaviors in experimental models—most notably Transient Receptor Potential Vanilloid-1 (TRPV1), Transient Receptor Potential Ankyrin-1 (TRPA1), TRPV4, and voltage-gated sodium channel–dependent excitability [46–48]. Pharmacologic antagonism of TRPV1 and TRPA1 attenuates pancreatitis-associated pain and has been shown in preclinical studies to reduce the transition from recurrent acute to chronic pancreatic pain [46]. Translationally, the ongoing phase 1 Safety, Tolerability, and Dose Limiting Toxicity of Lacosamide in Patients with Painful CP (STTEPP) trial (NCT05603702) is evaluating add-on lacosamide, a voltage-gated sodium channel modulator, in patients with painful CP [46, 47, 49–52].

**Fig. 1:**
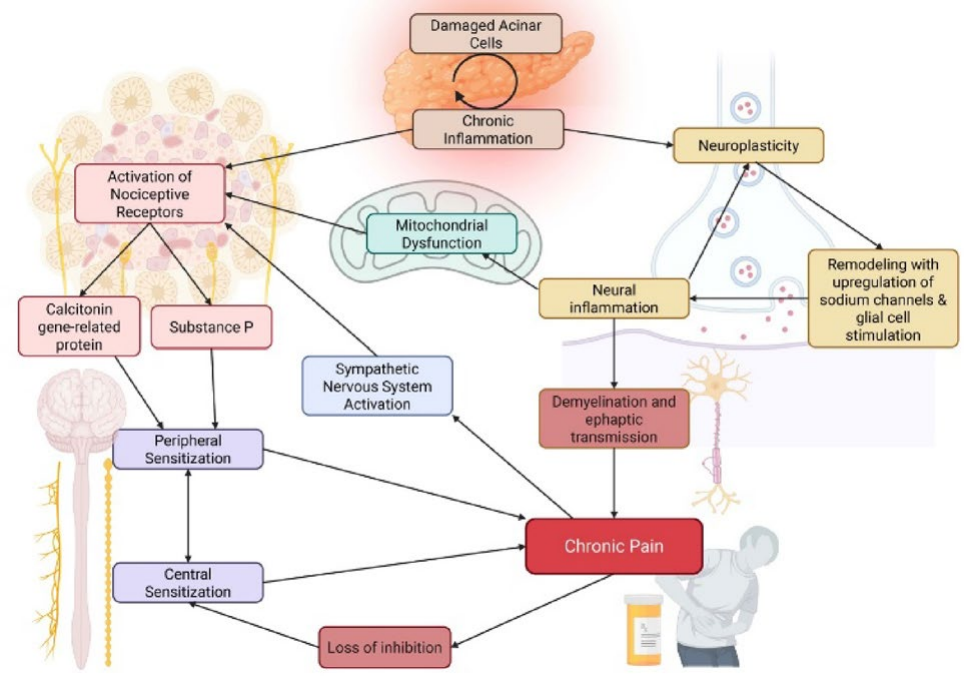
Proposed mechanisms underlying chronic pain in chronic pancreatitis (CP): Schematic overview of the multifactorial pathophysiology of chronic pain in CP. Recurrent pancreatic injury leads to acinar cell damage and persistent inflammation, resulting in activation of nociceptive afferent pathways. Release of neuropeptides, including substance P and calcitonin gene-related peptide, contributes to neural inflammation and peripheral sensitization. Ongoing inflammatory signaling promotes neuroplastic changes characterized by upregulation of sodium channels, glial cell activation, mitochondrial dysfunction, demyelination, and ephaptic transmission. These processes facilitate central sensitization, loss of inhibitory control, and sympathetic nervous system activation, collectively sustaining and amplifying chronic pain in CP

Persistent nociceptive and neuropathic signaling contributes to ascending sensitization within the central nervous system through activity-dependent synaptic potentiation [53]. N-methyl-D-aspartate (NMDA) receptors at afferent terminals respond to glutamatergic neurotransmission, permitting calcium influx [53]. Downstream effects include sustained hyperexcitability of central nociceptive neurons and strengthening of synaptic signaling, analogous to long-term potentiation in learning and memory [33, 53]. Mechanisms underlying this potentiation include upregulation of low-voltage–activated (T-type) calcium channels and NMDA receptors, along with downregulation of potassium channels, thereby promoting sustained cation influx and neuronal hyperexcitability [26, 43, 53]. Further investigation of these mechanisms is needed to clarify how central sensitization pathways relate to heterogeneous clinical pain trajectories in CP [30] (Fig. 1).

#### Transient receptor potential channels

Ion channels within the TRP family are key mediators of nociceptive signaling and represent attractive therapeutic targets [50, 54]. These channels participate in the initial detection of noxious stimuli, including inflammatory mediators, heat, and mechanical stress [54, 55]. Several TRP subtypes including TRPV1, TRPA1, TRPV4, and calcium-selective TRP Canonical-5/6 (TRPC5 and TRPC6) contribute to generator potentials and neuronal excitability [54]. Activation of TRP channels at nociceptive terminals induces calcium influx and subsequent activation of the mitogen—activated protein kinase (MAPK) pathway [56]. Downstream signaling includes Extracellular Signal-Regulated Kinase-1/2 (ERK1 and ERK2) and nuclear factor-kappa B (NF-κB) activation, leading to upregulation of pro-inflammatory cytokines [56, 57]. Release of neuropeptides such as substance P results in positive feedback amplification of local inflammatory signaling and mast cell activation, reinforcing neuroinflammation [28, 43, 58, 59]. Calcium influx further promotes peripheral sensitization through protein kinase C (PKC) activation and enhanced TRP channel expression [57].

TRPV4 activation has also been linked to Ak strain transforming (Akt) pathway signaling, which regulates cellular survival and apoptosis pathways in various disease contexts [54]. Emerging evidence suggests mechanosensitive signaling involving Piezo1–TRPV4 interactions in pancreatic stellate cells may influence stromal remodeling and inflammatory signaling in pancreatic disease [60, 61]. While TRP channel dysregulation has also been implicated in neurodegenerative disorders such as Alzheimer’s disease and amyotrophic lateral sclerosis, their central role in nociceptive and peripheral neuropathic pain mechanisms makes them particularly compelling targets in CP [46, 58]. Preclinical studies further suggest that TRP antagonism may attenuate the progression from recurrent acute pancreatitis to chronic pain phenotypes [46].

#### Purinergic and acid sensing ion channels

Purinergic receptors represent another class of ion channels involved in calcium-mediated nociceptive signaling [62]. Many downstream effects overlap with TRP-mediated pathways, including activation of MAPK, ERK1/2, Akt, and PKC signaling cascades, thereby reinforcing local inflammation, direct nociception, and peripheral sensitization [62–64]. Unlike TRP channels, purinergic receptors are adenine triphosphate (ATP) dependent, and their effects on neuronal excitation are influenced by the magnitude and duration of inflammatory signaling [62, 65]. In addition to their role in direct nociception, purinergic receptors are expressed on microglia and other pro -inflammatory immune cells [63]. Activation of macrophages and lymphocytes through purinergic signaling promotes reactive oxygen species generation and cytokine release, while microglial activation contributes to neuroplasticity and central sensitization [62, 63]. Given their involvement in both nociceptive and neuroinflammatory processes, therapeutic strategies targeting purinergic receptors may warrant investigation across nociceptive, neuropathic, and centrally sensitized pain phenotypes in CP [53, 64, 66].

Acid-sensing ion channels (ASICs) are proton-gated sodium channels within the epithelial sodium channel (ENaC) family that mediate inflammatory pain in the setting of tissue acidosis associated with inflammation, ischemia, or injury [67]. Beyond their role in peripheral sensitization and hyperalgesia through cation influx, ASICs also participate in synaptic potentiation between peripheral and central sensory neurons and are thought to contribute to central sensitization mechanisms [67]. While ASICs have been implicated in visceral hypersensitivity in other gastrointestinal inflammatory conditions, their specific contribution to CP pain has not been directly investigated and represents an important knowledge gap amenable to omics-based approaches [68].

#### Voltage-gated ion channels

Voltage-gated sodium channels (VGSCs) and voltage-gated calcium channels (VGCCs) are critical regulators of depolarization, action potential propagation, neurotransmitter release, and neuronal excitability [44]. At sensory terminals, these channels regulate generator potential amplitude and influence activation thresholds required for nociceptive signal transmission [69]. Sodium channels concentrated at the nodes of Ranvier facilitate rapid propagation of nociceptive signals along the axon toward synaptic terminals [44]. Modulation of VGSCs plays an important role in peripheral and central sensitization, contributing to hypersensitivity to noxious stimuli and the development of neuropathic components of chronic CP pain [70]. The precise mechanisms governing VGSC modulation in CP remain incompletely understood [2, 71]. Recent clinical translation of VGSC targeting includes U.S. Food and Drug Administration approval of suzetrigine (Journavx), a first -in-class selective NaV1.8 inhibitor, for moderate-to-severe acute pain in adults, providing proof-of-concept that peripheral sodium channel blockade can yield clinically meaningful analgesia [72, 73].

Low-voltage–activated calcium channel density is increased in certain neuropathic pain models, including diabetic neuropathy and nerve constriction models [69]. In preclinical pancreatitis models, Cav3.2 T-type calcium channels have been shown to mediate pancreatic nociception, with selective T-channel inhibition suppressing referred hyperalgesia [74, 75]. High-voltage-activated calcium channel antagonists have also demonstrated efficacy in attenuating abdominal hyperalgesia and inflammatory responses in acute pancreatitis [76, 77].

### Metabolic dysfunction and mitochondrial stress

Metabolic and mitochondrial stress are increasingly implicated in pancreatitis pathobiology and pain sensitization; however, their specific contribution to the transition from acute to CP-associated pain and their interaction with ion-channel remodeling and genetic programs remains incompletely defined [30, 78–80]. Metabolic dysfunction contributes to neuronal impairment and the pathophysiology of both nociceptive and neuropathic pain [19]. Improved characterization of metabolic pathways underlying chronic pain may enable identification of metabolic phenotypes that better stratify CP patients into mechanism—informed treatment groups. Bioenergetic dysfunction also modulates ion-channel activity and neuronal excitability, potentially facilitating long-term potentiation of nociceptive synapses [44, 81].

Damage-associated molecular patterns (DAMPs) and local inflammation promote neuroinflammation and oxidative stress, directly impairing mitochondrial function [3, 82, 83]. Although neurons exhibit high basal production of reactive oxygen and nitrogen species (ROS and RNS), mitochondrial dysfunction may exacerbate this production, leading to redox imbalance [84, 85]. Excess ROS and RNS further reinforce nociceptive and neuropathic signaling through activation of canonical inflammatory and apoptotic pathways [84]. These processes may be amplified by aging, alcohol use, and smoking [86].

Calcium homeostasis represents a critical metabolic function coordinated by mitochondria and the endoplasmic reticulum (ER) [87]. The ER serves as the primary intracellular calcium reservoir and regulates calcium exchange with mitochondria through mitochondria-associated membranes (MAMs) [87, 88]. Prolonged ER stress can disrupt calcium homeostasis, increasing neuronal excitability and further impairing mitochondrial function [87, 88]. Elevated intracellular Ca^2+^ alters gene expression and promotes synaptic vesicle fusion, enhancing release of nociceptive neurotransmitters [89, 90].

**Fig. 2:**
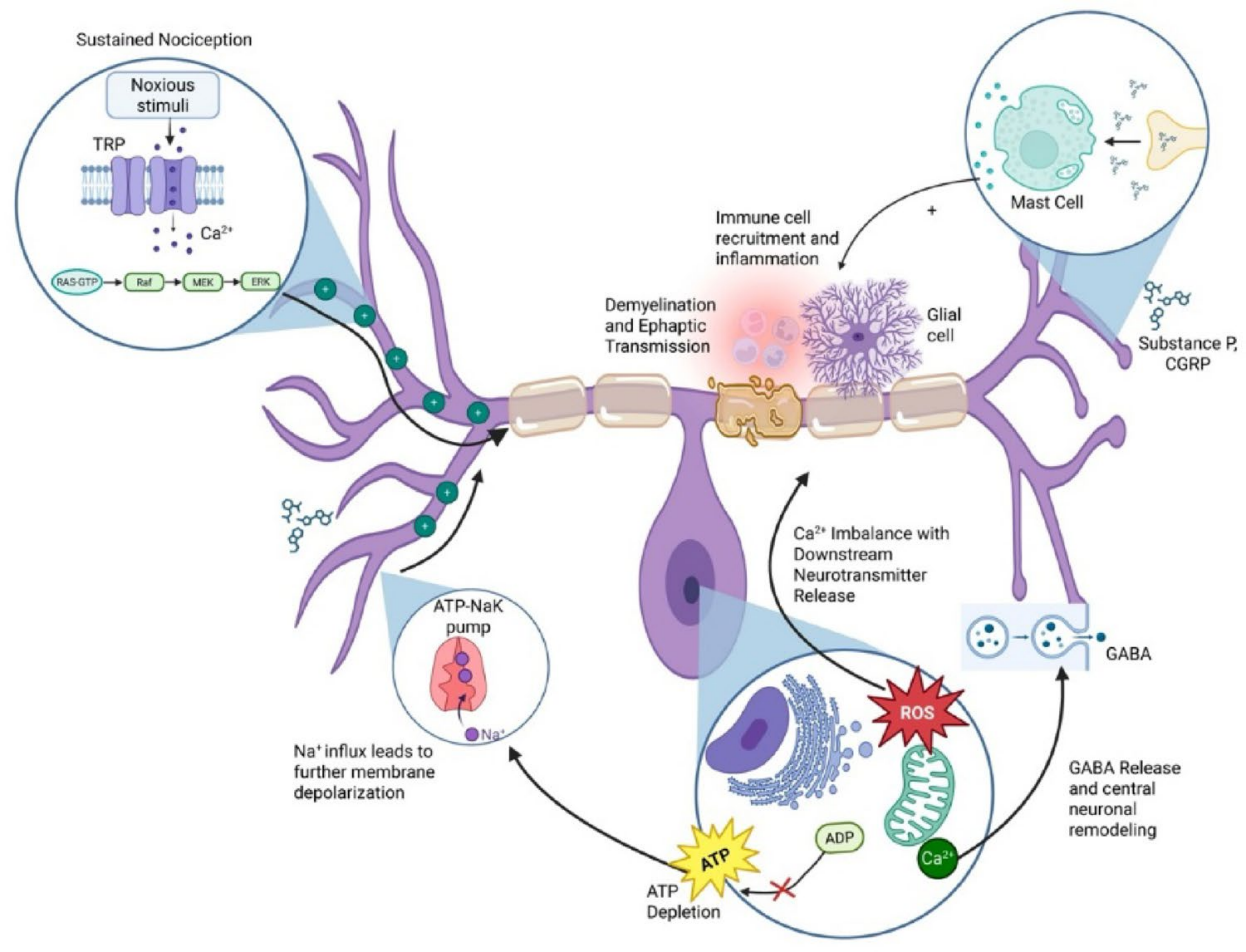
Cellular mechanisms driving neuroinflammation, neuroexcitability, and central sensitization in chronic pancreatitis pain. Noxious stimuli activate transient receptor potential (TRP) channels on sensory nerve terminals, initiating calcium influx and downstream MAPK signaling that sustains nociceptive transmission. Neuropeptide release (substance P, CGRP) triggers mast cell degranulation and immune cell recruitment, promoting neuroinflammation and demyelination with ephaptic transmission. Metabolic dysfunction including ATP depletion, reactive oxygen species (ROS) generation, and impaired Na⁺/K⁺-ATPase activity causes membrane depolarization and neuronal hyperexcitability

Mitochondrial ATP depletion increases neuronal excitability by impairing ATP-dependent homeostatic pumps, including Na⁺/K⁺-ATPase and Ca^2+^-handling mechanisms, thereby promoting membrane depolarization and enhanced neurotransmitter release [91]. In parallel, ATP-sensitive potassium (KATP) channels function as metabolic brakes on excitability; reduced KATP activity has been associated with enhanced nociceptive signaling in neuropathic pain models [92]. Collectively, bioenergetic impairment, redox imbalance, disrupted ER–mitochondrial Ca^2+^ signaling, and impaired ATP-dependent ionic homeostasis can alter membrane potential and ion-channel gating, lower firing thresholds, and amplify both nociceptive and neuropathic signaling in peripheral afferents and central circuits (Fig. 2) [85, 87, 91]. The extent to which these metabolic perturbations contribute to heterogeneity in pain phenotypes among patients with CP remains an important gap in the literature.

Calcium dysregulation enhances neurotransmitter release and synaptic potentiation, driving central neuronal remodeling and sensitization. Plus signs (+) indicate excitatory or amplifying effects. Abbreviations: ATP, adenosine triphosphate; CGRP, calcitonin gene-related peptide; ERK, extracellular signal-regulated kinase; GABA, gamma-aminobutyric acid; MAPK, mitogen-activated protein kinase; MEK, MAPK kinase; ROS, reactive oxygen species; TRP, transient receptor potential.

## Genomics and predisposition of different pain phenotypes

Hereditary variants constitute a key risk category within the Toxic-metabolic, Idiopathic, Genetic, Autoimmune, Recurrent acute pancreatitis, Obstructive (TIGAR-O) classification schema for CP [93]. Recognized pathogenic mechanisms underlying hereditary pancreatitis include premature intraductal trypsinogen activation, induction of endoplasmic stress within acinar cells, and increased intraductal calcium concentrations [93–96]. Recurrent acute pancreatitis and progression to CP result in sustained inflammation, direct nociceptive activation, and feed-forward mechanisms that promote chronic nociceptive and neuropathic pain, ultimately contributing to central sensitization [30]. Although neuropathic mechanisms are central to chronic pain in CP, limited data exist regarding the contribution of genetic channelopathies to pain phenotypes or their interaction with variants implicated in the sentinel acute pancreatitis event [97]. TRPV6 variants have been identified in association with established hereditary pancreatitis genes including PRSS1, CFTR, SPINK1, CTRC, and CPA1 in a substantial proportion of early-onset CP cases [98]. Loss-of-function TRPV6 mutations impair calcium influx into acinar cells, resulting in elevated intraductal calcium concentrations and increased CP susceptibility [98]. Variants in epithelial sodium channel (ENaC) genes, including SCNN1B and SCNN1G, as well as polymorphisms in TNFRSF1A, have also been implicated in chronic pain syndromes and may represent modifiers of pain risk in CP [99]. While SCN9A polymorphisms have been associated with altered pain perception across multiple conditions including pancreatitis, dedicated studies examining nociceptive ion channelopathies in relation to specific CP pain phenotypes remain limited [100].

Future studies integrating nociceptive ion channel dysregulation with established CP genotypes may enable more precise application of ion channel–targeted therapeutics. Expanded genomic analyses incorporating gene–gene interactions could further refine biologically informed pain phenotyping in CP. A comprehensive genotype–phenotype study by Dunbar et al. recently evaluated genetic associations with distinct pain patterns in CP [21]. The investigators identified four principal gene systems: (1) pancreas injury and stress pathways (e.g., REG gene cluster, CTRC, NEURL3, and HSF2), (2) neuronal development and axon guidance pathways (including SNPO, RGMA, MAML1, and DOK6, a component of the RET signaling complex), (3) genes associated with psychiatric stress—related pathways (TMEM65, RBFOX1, and ZNF3858D), and (4) BDNF-related neuropathic signaling pathways (including SYNPR, NTF3, and RBFOX1) [21]. These gene systems were associated with constant, severe, and constant -severe pain phenotypes [21]. Integrating such genomic classifications with metabolic and neurophysiologic domains may yield a more comprehensive understanding of mechanistic pain heterogeneity in CP and support development of phenotype -specific therapeutic strategies.

## Current clinical pain phenotypes and measurement

Commonly used pain assessments such as the Brief Pain Inventory, numeric rating scales, and the visual analog scale are low-dimensional and insufficient to capture the heterogeneity of CP pain [16, 101]. Prior work has classified CP pain according to underlying neurophysiologic mechanisms [5, 15]. Within this framework, descriptors such as “burning” or “stinging” align with neuropathic mechanisms, whereas “aching” or “sore” reflect nociceptive processes [5]. The PROMIS nociceptive and neuropathic pain instruments have been applied clinically to phenotype CP pain, with over two -thirds of patients categorized as nociceptive, neuropathic, or mixed. Notably, 24% remained unclassifiable, underscoring the need for more precise, mechanism-informed phenotyping [5].

Central sensitization represents a distinct mechanistic domain, clinically characterized by secondary hyperalgesia [30]. Pain arising from peripheral or central sensitization without clear inflammation or somatosensory injury is increasingly termed nociplastic pain [102, 103]. The COMPAT tool was developed to incorporate mechanistic pain evaluation in CP alongside quality of life, pain distribution, temporal pattern, opioid use, and related modifiers [40, 101]. Current validation standards for pain instruments recommend multidimensional assessment, including pain intensity and quality, physical function and health-related quality of life, emotional distress, longitudinal measurement, and adverse events [16].

Existing tools address these domains to varying extents (Table 1).

**Table 1:** Existing pain instruments and core domains of pain assessed

	Core domains assessed			
Tools	Pain	Physical	Emotional	Global
*General unidimensional assessments*				
Visual analog scale	x			
Numeric rating scale	x			
Categorical pain assessment	x			
*General multidimensional assessments*				
McGill pain assessment	x	x		
PainDetect questionnaire	x			
Brief pain inventory	x	x	x	
Pain disability index		x	x	
*Instruments designed for pancreatitis associated chronic pain*				
COMPAT	x			
PANQOLI	x	x	x	x
Izbicki pain scale	x	x		
PROMIS instruments	x	x	x	x
QST	x			

COMPAT, comprehensive pain assessment tool; PANQOLI, pancreatitis quality of life instrument; PROMIS, patient-reported outcomes measurement information system; QST, quantitative sensory testing

*Domain definitions:* Pain intensity/qualities = severity and/or sensory descriptors (e.g., neuropathic-like features depending on instrument). Physical function/disability = interference with activities, work, role function, or disability indices. Emotional/affect = mood-related impact, distress, or affective descriptors tied to pain. Global health/QoL = overall health status and/or quality-of-life summaries (disease-specific or generic)

BPI, brief pain inventory; COMPAT, comprehensive pain assessment tool; MPQ, McGill pain questionnaire; NRS, numeric rating scale; PANQOLI, pancreatitis quality of life instrument; PDI, pain disability index; PROMIS, patient-reported outcomes measurement information system; QST, quantitative sensory testing; VAS, visual analog scale

This table summarizes commonly used pain instruments and the key domains they capture, including pain severity/qualities, physical function, emotional impact, and global health. Coverage varies substantially across tools, underscoring the need for harmonized, multidimensional phenotyping in chronic pancreatitis pain research

Guidelines also emphasize accounting for alcohol -associated chronic pain, given its distinct cognitive and psychosocial modifiers [104]. Medication exposure is incorporated in several scoring systems, including the Izbicki pain score and the Multiple risk factors—Alcohol, Nicotine, Nutritional factors, Hereditary factors, Efferent duct factors, Immunological factors, and Miscellaneous/Metabolic factors (M-ANNHEIM) grading system [16]. Additional tools used to refine pain phenotypes including QST, the Hospital Anxiety and Depression Scale (HADS), and the Pain Catastrophizing Scale (PCS) [15, 16, 105, 106]. Future iterations of CP pain assessment may integrate such clinical phenotypes with biologic markers through multimodal omics-based approaches.

Psychiatric and behavioral comorbidities further modulate pain perception. A recent cross-sectional study demonstrated associations between depression, anxiety, sleep disturbance, low activity levels, and both intermittent and constant pain patterns [107]. Comorbidity burden, active smoking, and pain-related disability also significantly affect pain-related quality of life [8]. Emerging evidence suggests potential immunomodulatory and genetic links between psychiatric conditions and CP, and genetic loci associated with anxiety and depression have been correlated with constant severe pain phenotypes [32, 108]. However, most data are cross-sectional, leaving the temporal and causal relationships between psychiatric conditions and pain severity unresolved.

Given this interplay, cognitive behavioral therapy and pharmacologic interventions are recommended within the World Health Organization analgesic ladder before opioid escalation in selected patients [29]. Chronic pain itself contributes to structural and functional reorganization of the central nervous system, including diminished descending inhibitory control and amplification of central sensitization [109]. In 2019, the International Association for the Study of Pain formally defined chronic primary pain as a category encompassing disorders such as fibromyalgia, complex regional pain syndrome, and irritable bowel syndrome (IBS) [103, 110]. Because centralized mechanisms can influence symptom reporting and alter QST measures, future CP pain phenotyping and instrument validation should account for comorbid centralized pain disorders (e.g., fibromyalgia, IBS) as potential modifiers of pain burden, opioid exposure, and quality-of-life outcomes [38, 103, 111–114].

### Advancing precision pain management through integrated multi-omics and computational modeling

**Fig. 3:**
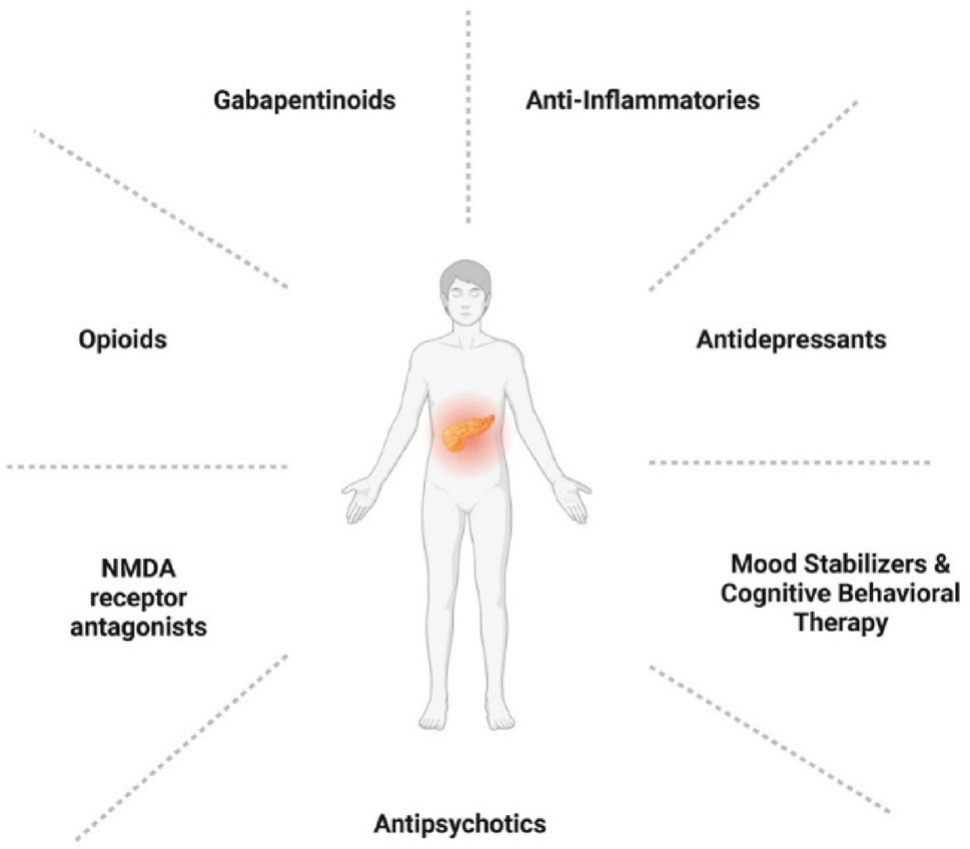
Current treatment approaches include gabapentinoids, anti-inflammatories, antidepressants, opioids, N-methyl-D-aspartate (NMDA) receptor antagonists, antipsychotics, mood stabilizers, and cognitive behavioral therapy. The heterogeneity in pain response across these modalities underscores the need for mechanism -based patient stratification to optimize individualized treatment selection. Abbreviations: NMDA, N-methyl-D-aspartate receptor

The marked biologic and clinical heterogeneity of CP pain necessitates a precision framework capable of aligning patients with mechanism-targeted therapies. However, existing management approaches span multiple pharmacologic and behavioral modalities and are often applied empirically, with variable benefit across patients (Fig. 3).

**Fig. 4:**
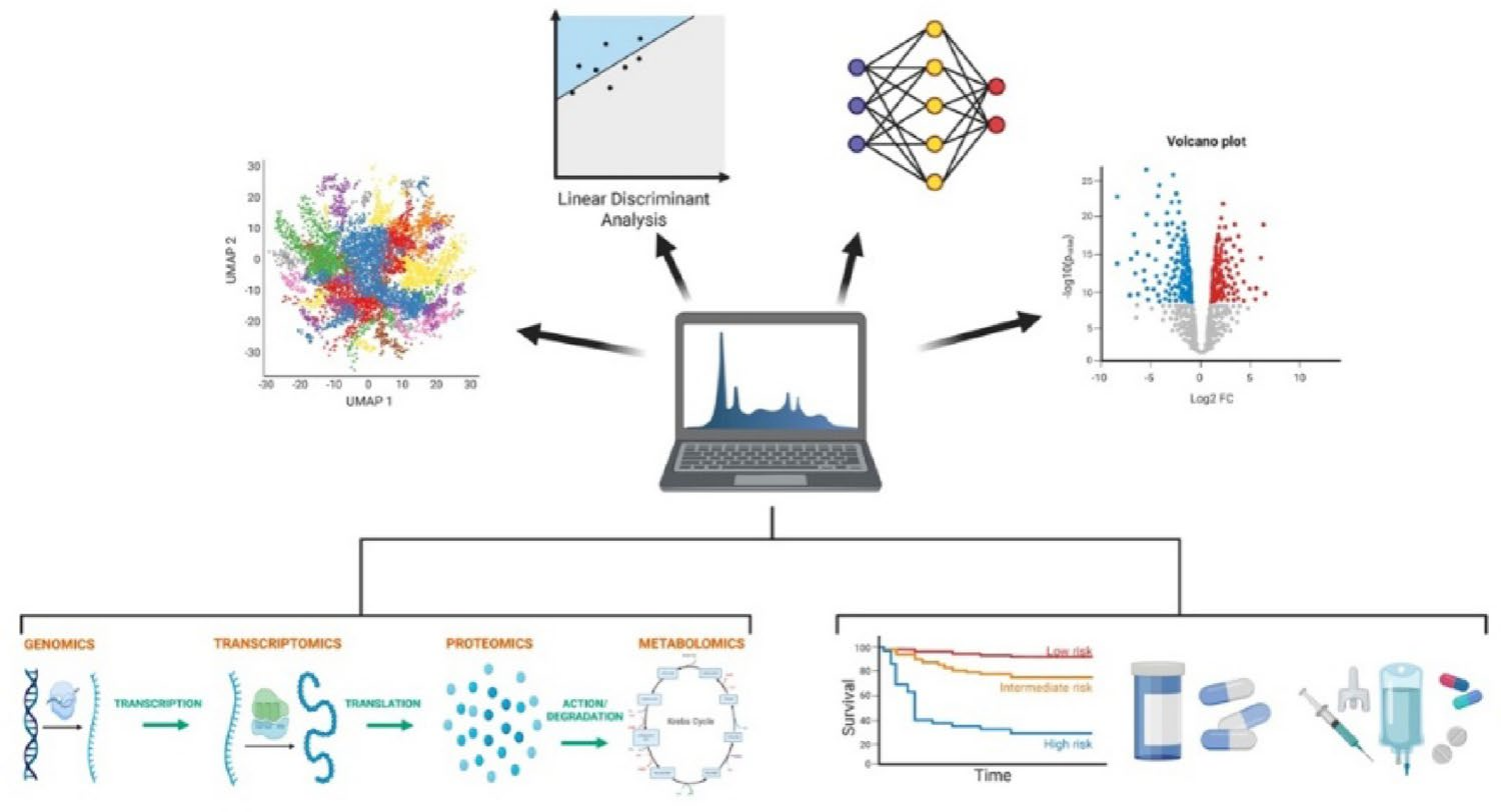
Integrated multi-omics framework for precision pain management in chronic pancreatitis. Multi-omics data, including genomics, transcriptomics, proteomics, and metabolomics, are integrated with clinical phenotypes to generate high-dimensional datasets. Computational approaches such as dimensionality reduction, linear discriminant analysis, neural networks, and differential expression analysis enable identification of molecular signatures and patient subgroups. These integrated models can stratify patients by risk and predict clinical outcomes, ultimately guiding individualized therapeutic selection

A multi-omics strategy of integrating genomics, transcriptomics, proteomics, and metabolomics offers a pathway toward defining biologically grounded pain phenotypes that extend beyond symptom-based classification [23, 42]. Similar to precision oncology paradigms in pancreatic cancer, correlating standardized clinical pain phenotypes with molecular signatures may uncover mechanistic drivers of persistent pain and identify predictive biomarkers of therapeutic response [115] (Fig. 4).

Linking genotype with downstream transcriptomic, proteomic, and metabolomic profiles enables construction of high-dimensional models that more accurately capture biologic variation across patients. Such integrative approaches move beyond isolated molecular associations and instead characterize coordinated pathway-level dysregulation. These refined endotypes could inform biomarker discovery, support predictive enrichment strategies for clinical trials, and ultimately guide individualized therapy selection [42, 116].

Operationalizing this framework requires anchoring computational integration to biologically plausible systems. The four genome-based networks identified by Dunbar et al. provide a foundation for embedding genetic architecture within previously described clinical pain phenotypes [21, 32]. Expanding this work to interrogate gene–gene interactions particularly between pain-associated variants and canonical hereditary pancreatitis genes may clarify how inherited susceptibility intersects with pain chronification. Downstream transcriptomic and proteomic profiling can further delineate functional consequences of these variants, enabling identification of circulating or tissue-based biomarkers that serve as clinically accessible surrogates for genomic risk [25]. If future -omics investigations identify therapeutics targeting BDNF-mediated neuropathic mechanisms, circulating BDNF could function as a predictive biomarker for treatment response. Similarly, metabolomic profiling may reveal metabolic intermediates that both illuminate altered bioenergetic or inflammatory pathways and provide measurable signatures predictive of analgesic efficacy. Coordinated integration of biomarkers, such as BDNF, with genomic architecture, standardized pain phenotypes, and neurophysiologic measures is essential to constructing biologically coherent and clinically actionable pain endotypes [5, 24, 116].

Emerging data already suggest translational potential. A recent study identified TGFβ-1 and serum gp130 as candidate biomarkers distinguishing nociceptive and mixed pain phenotypes, respectively [25]. Given the growing recognition of neuropathic mechanisms in CP, proteomic mapping of peripheral and central neuronal ion channel expression may further expand therapeutic targets and stratification strategies [117]. However, translating such signals into clinical decision tools requires integrated computational modeling capable of fusing multi-omics layers with longitudinal clinical data.

Advanced bioinformatics approaches enable synthesis of predictive models from high-dimensional datasets to estimate therapeutic response and disease trajectory [118]. While multi-omics integration has been most extensively applied in oncology to guide chemotherapy selection, analogous strategies are increasingly feasible in chronic pain research [119]. Broadly, integration frameworks include supervised learning models that predict predefined outcomes (e.g., treatment response), unsupervised methods that identify latent patient clusters, and hybrid or network-based approaches that map pathway interactions across molecular layers [120, 121]. Deep learning architectures can capture nonlinear, multiscale interactions, whereas dimensionality-reduction and factorization methods help mitigate overfitting while preserving biologically meaningful structure. Clustering techniques may reveal novel mechanistic subgroups, potentially redefining CP pain phenotypes beyond traditional classifications.

Nonetheless, substantial challenges accompany multi-omics modeling. Heterogeneity in data type, scale, and measurement platforms increases risk of technical bias and spurious associations. The dimensionality of omics data relative to sample size amplifies overfitting risk, and model interpretability remains critical for clinical adoption [122]. Accordingly, rigorous cross-cohort harmonization, transparent feature selection, external validation, and biologically anchored interpretation are essential to ensure reproducibility and translational relevance.

Leveraging multicenter biobanks and collaborative cohorts such as the PROspective Evaluation of Chronic Pancreatitis for EpidEmiologic and Translational StuDies (PROCEED), International Study group of Pediatric Pancreatitis: In search for a cuRE (INSPPIRE), and Diabetes Related to Acute Pancreatitis and its Mechanisms (DREAM) provides a scalable infrastructure for this work [123–125]. Key priorities include harmonization of common data elements, adoption of standardized and repeatedly measured patient-reported pain outcomes, and expansion of longitudinal paired biosampling across biologic compartments. Such efforts would enable integrated neuroimmune mapping, validation of mechanistic endotypes, and identification of responder profiles across diverse patient populations.

Integration of biologic data into clinical workflows also demands careful attention to data preprocessing and implementation science. Missingness must be characterized mechanistically and addressed using methods that preserve longitudinal trajectories. Noise reduction and normalization strategies must retain pathway-level signals while minimizing artifact. Model outputs should be interpretable, anchored to known mechanisms, and contextualized alongside clinical measures to generate testable hypotheses for prospective validation. Finally, precision pain models must demonstrate fairness across demographic and etiologic subgroups, scalability across sites, and robust privacy safeguards to support real-world deployment [122, 126].

Collectively, a rigorously constructed, multi-omics–integrated computational framework offers a pathway from descriptive pain phenotyping toward biologically stratified, mechanism-guided therapy in CP. By linking molecular architecture with clinical trajectories and therapeutic response, precision pain management can evolve from empiric trial-and-error toward a predictive and personalized paradigm.

## Challenges and limitations

Despite the promise of multi-omics integration, substantial barriers must be addressed before precision pain management can be realized in CP. First, cohort size requirements for adequately powered multi-omics studies are considerable; the A2CPS program, for example, is enrolling 2,800 participants to develop predictive biosignatures for chronic postsurgical pain, and similar or larger cohorts would likely be required for CP-specific biomarker discovery and validation [41]. Given the relatively low incidence of CP, achieving such sample sizes will necessitate sustained multicenter collaboration over extended timeframes.

Second, methodological heterogeneity poses a significant challenge. Differences in omics platforms, data preprocessing pipelines, normalization strategies, and analytical frameworks complicate cross-study comparisons and limit reproducibility [127, 128]. The field remains dominated by studies integrating only pairs of omics layers, and computational tools for comprehensive multi-layer integration remain underdeveloped [127]. Moreover, most computational findings lack experimental validation, and standardized frameworks for cross-study comparison are lacking.

Third, cost and resource constraints represent practical barriers to implementation. Multi-omics profiling requires specialized infrastructure, technical expertise, and substantial financial investment that may not be available at all clinical centers [129, 130]. The economic viability of routine multi-omics testing in clinical practice remains unestablished, and achieving "economically viable, environmentally sustainable, and universally accessible" precision pain solutions will require strategically streamlined methods.

Fourth, clinical interpretability and implementation present ongoing challenges. Translating high-dimensional omics data into actionable clinical decisions requires interpretable models that clinicians can understand and trust [126, 131]. Health-care professionals require continued exposure to precision medicine approaches to be confident in applying them to practice [132]. Additionally, regulatory pathways for multi-omics-based clinical decision tools remain undefined.

Finally, the heterogeneity of CP itself encompassing diverse etiologies, variable disease duration, and overlapping pain mechanisms may limit the generalizability of biomarker signatures across patient subgroups. Whether molecular signatures identified in one CP population will translate to patients with different etiologies (e.g., alcoholic vs. hereditary vs. idiopathic CP) remains uncertain. Rigorous external validation across demographically and etiologically diverse cohorts will be essential before clinical implementation. Acknowledging these challenges, the framework outlined in this review represents a long-term research agenda rather than an immediately achievable clinical paradigm. Realizing this vision will require sustained investment in collaborative biobanking, harmonization of data collection standards, development of interpretable computational models, and prospective validation studies demonstrating clinical utility and cost-effectiveness.

## Future directions

Substantial heterogeneity characterizes the pain experience, underlying biologic mechanisms, and therapeutic response in CP [16, 21, 30]. Although the WHO analgesic ladder provides a pragmatic framework for treatment escalation, it does not account for mechanistic diversity or enable prediction of individual response [3, 7]. Advancing CP pain management therefore requires biologically informed patient stratification capable of aligning specific pain endotypes with targeted interventions [42, 117, 126].

Progressive CP is associated with central sensitization and impaired descending inhibitory control, suggesting that mechanism-based intervention earlier in the disease course may mitigate chronification and neural remodeling [31]. Earlier identification of high-risk phenotypes will depend on clinically accessible biomarkers that link measurable protein or metabolite expression to implicated genomic and metabolic pathways [5, 133]. Integrating such biomarkers with standardized clinical phenotyping frameworks may allow prospective prediction of therapeutic responsiveness rather than retrospective adjustment after treatment failure [116, 126].

Modern machine learning and artificial intelligence platforms provide the analytical infrastructure to integrate multi -omics data with neurophysiologic and imaging modalities [41]. Objective biologic signatures could complement QST, electroencephalography, and functional magnetic resonance imaging, enhancing mechanistic classification beyond symptom-based tools alone [12, 134, 135]. Incorporating biomarkers associated with stress-response and psychiatric vulnerability pathways may further refine stratification and guide integration of cognitive behavioral therapy within individualized treatment plans [29].

Ultimately, the future of CP pain management will likely move toward multidimensional phenotypes that synthesize integrated omics data, neurophysiology, psychiatric comorbidity, and anatomic considerations that guide endoscopic or surgical therapy. Such a framework supports earlier intervention, rational therapeutic sequencing, and reduction of empiric opioid escalation.

## Conclusions

CP pain arises from complex, interacting genetic, environmental, metabolic, inflammatory, and neurophysiologic mechanisms that drive substantial inter-patient variability in clinical presentation and therapeutic response. Metabolic dysregulation including ATP depletion, oxidative stress, and calcium imbalance intersects with neuroimmune signaling and ion-channel modulation to promote peripheral sensitization, central sensitization, and structural and functional reorganization of central pain pathways. These convergent processes generate distinct yet overlapping pain phenotypes encompassing nociceptive, neuropathic, and nociplastic mechanisms.

Although genetic susceptibility and environmental exposures clearly influence pain burden, their integration with downstream neurophysiology and druggable target expression remains incompletely defined. By synthesizing evidence across human cohorts and preclinical models, this review outlines a translational framework that links standardized pain phenotyping with integrated multi-omics to enable biomarker-guided, precision analgesic and procedural decision-making.

Multi-omics integration offers a promising strategy to resolve current mechanistic gaps, identify actionable biomarkers, and associate molecular signatures with reproducible clinical trajectories. When coupled with rigorously validated machine learning models, these approaches have the potential to predict therapeutic response to mechanism -targeted interventions such as selective sodium channel inhibitors or neuroimmune-directed therapies and to shift CP pain management from reactive, symptom-based escalation toward a predictive, biologically grounded paradigm.

## Data Availability

No datasets were generated or analysed during the current study.

## References

[1] Yadav D, Lowenfels AB. The epidemiology of pancreatitis and pancreatic cancer. Gastroenterology. 2013;144(6):1252–61. 10.1053/j.gastro.2013.01.068. 23622135 10.1053/j.gastro.2013.01.068 PMC3662544

[2] Thierens N, Verdonk RC, Lohr JM, van Santvoort HC, Bouwense SA, van Hooft JE. Chronic pancreatitis. Lancet. 2025;404(10471):2605–18. 10.1016/S0140-6736(24)02187-1. 39647500 10.1016/S0140-6736(24)02187-1

[3] Singh VK, Yadav D, Garg PK. Diagnosis and management of chronic pancreatitis: a review. JAMA. 2019;322(24):2422–34. 10.1001/jama.2019.19411. 31860051 10.1001/jama.2019.19411

[4] Vege SS, Chari ST. Chronic pancreatitis. N Engl J Med. 2022;386(9):869–78. 10.1056/NEJMcp1809396. 35235728 10.1056/NEJMcp1809396

[5] Saloman JL, Conwell DL, Fogel E, Vege SS, Li L, Li S, et al. Characterizing mechanism-based pain phenotypes in patients with chronic pancreatitis: a cross-sectional analysis of the PROspective Evaluation of Chronic Pancreatitis for EpidEmiologic and Translational StuDies. Pain. 2023;164(2):375–84. 10.1097/j.pain.0000000000002710. 36149018 10.1097/j.pain.0000000000002710 PMC9726990

[6] Gardner TB, Kennedy AT, Gelrud A, Banks PA, Vege SS, Gordon SR, et al. Chronic pancreatitis and its effect on employment and health care experience: results of a prospective American multicenter study. Pancreas. 2010;39(4):498–501. 10.1097/MPA.0b013e3181c5c693. 20118821 10.1097/MPA.0b013e3181c5c693

[7] Drewes AM, Bouwense SAW, Campbell CM, Ceyhan GO, Delhaye M, Demir IE, et al. Guidelines for the understanding and management of pain in chronic pancreatitis. Pancreatology. 2017;17(5):720–31. 10.1016/j.pan.2017.07.006. 28734722 10.1016/j.pan.2017.07.006

[8] Machicado JD, Amann ST, Anderson MA, Abberbock J, Sherman S, Conwell DL, et al. Quality of life in chronic pancreatitis is determined by constant pain, disability/unemployment, current smoking, and associated co-morbidities. Am J Gastroenterol. 2017;112(4):633–42. 10.1038/ajg.2017.42. 28244497 10.1038/ajg.2017.42 PMC5828017

[9] de Rijk FEM, van Veldhuisen CL, Kempeneers MA, Issa Y, Boermeester MA, Besselink MG, et al. Quality of life in patients with definite chronic pancreatitis: a nationwide longitudinal cohort study. Am J Gastroenterol. 2023;118(8):1428–38. 10.14309/ajg.0000000000002266. 36996496 10.14309/ajg.0000000000002266

[10] van der Gaag NA, van Gulik TM, Busch OR, Sprangers MA, Bruno MJ, Zevenbergen C, et al. Functional and medical outcomes after tailored surgery for pain due to chronic pancreatitis. Ann Surg. 2012;255(4):763–70. 10.1097/SLA.0b013e31824b7697. 22418009 10.1097/SLA.0b013e31824b7697

[11] van Veldhuisen CL, Leseman CA, De Rijk FEM, Marques-Antunes J, Ausania F, Belyaev O, et al. Surgery for chronic pancreatitis across Europe (ESCOPA): prospective multicentre study. Br J Surg. 2025;112(4):znaf068. 10.1093/bjs/znaf068. 40296656 10.1093/bjs/znaf068 PMC12038157

[12] Buscaglia JM, Chang L. Pain phenotypes in chronic pancreatitis: beginning to fine-tune our approach to treatment. Clin Gastroenterol Hepatol. 2022;20(1):28–30. 10.1016/j.cgh.2020.12.032. 33387669 10.1016/j.cgh.2020.12.032

[13] Wilcox CM, Yadav D, Ye T, Gardner TB, Gelrud A, Sandhu BS, et al. Chronic pancreatitis pain pattern and severity are independent of abdominal imaging findings. Clin Gastroenterol Hepatol. 2015;13(3):552–60. 10.1016/j.cgh.2014.10.015. (**quiz e28–9**). 25424572 10.1016/j.cgh.2014.10.015 PMC5019545

[14] Van Veldhuisen CL, Leseman CA, De Rijk FEM, Dekker EN, Wellens MJ, Michiels N, et al. Nationwide outcome of tailored surgery for symptomatic chronic pancreatitis based on pancreatic morphology: validation of the international guidelines. Ann Surg. 2025;281(4):655–63. 10.1097/SLA.0000000000006176. 38088187 10.1097/SLA.0000000000006176 PMC11888824

[15] Phillips AE, Afghani E, Akshintala VS, Benos PY, Das R, Drewes AM, et al. Pancreatic quantitative sensory testing to predict treatment response of endoscopic therapy or surgery for painful chronic pancreatitis with pancreatic duct obstruction: study protocol for an observational clinical trial. BMJ Open. 2024;14(3):e081505. 10.1136/bmjopen-2023-081505. 38514147 10.1136/bmjopen-2023-081505 PMC10961514

[16] Drewes AM, van Veldhuisen CL, Bellin MD, Besselink MG, Bouwense SA, Olesen SS, et al. Assessment of pain associated with chronic pancreatitis: an international consensus guideline. Pancreatology. 2021;21(7):1256–84. 10.1016/j.pan.2021.07.004. 34391675 10.1016/j.pan.2021.07.004

[17] Demir IE, Tieftrunk E, Maak M, Friess H, Ceyhan GO. Pain mechanisms in chronic pancreatitis: of a master and his fire. Langenbecks Arch Surg. 2011;396(2):151–60. 10.1007/s00423-010-0731-1. 21153480 10.1007/s00423-010-0731-1 PMC3026929

[18] Ceyhan GO, Demir IE, Maak M, Friess H. Fate of nerves in chronic pancreatitis: neural remodeling and pancreatic neuropathy. Best Pract Res Clin Gastroenterol. 2010;24(3):311–22. 10.1016/j.bpg.2010.03.001. 20510831 10.1016/j.bpg.2010.03.001

[19] Willemen H, Santos Ribeiro PS, Broeks M, Meijer N, Versteeg S, Tiggeler A, et al. Inflammation-induced mitochondrial and metabolic disturbances in sensory neurons control the switch from acute to chronic pain. Cell Rep Med. 2023;4(11):101265. 10.1016/j.xcrm.2023.101265. 37944527 10.1016/j.xcrm.2023.101265 PMC10694662

[20] Szabo V, Csakany-Papp N, Gorog M, Madacsy T, Varga A, Kiss A, et al. Orai1 calcium channel inhibition prevents progression of chronic pancreatitis. JCI Insight. 2023. 10.1172/jci.insight.167645. 37227782 10.1172/jci.insight.167645 PMC10371343

[21] Dunbar EK, Greer PJ, Saloman JL, Albers KM, Yadav D, Whitcomb DC. Genetics of constant and severe pain in the NAPS2 cohort of recurrent acute and chronic pancreatitis patients. J Pain. 2025;27:104754. 10.1016/j.jpain.2024.104754. 39674387 10.1016/j.jpain.2024.104754 PMC12199748

[22] Lowe ME, Andersen DK, Caprioli RM, Choudhary J, Cruz-Monserrate Z, Dasyam AK, et al. Precision medicine in pancreatic disease-knowledge gaps and research opportunities: summary of a National Institute of Diabetes and Digestive and Kidney Diseases workshop. Pancreas. 2019;48(10):1250–8. 10.1097/MPA.0000000000001412. 31688587 10.1097/MPA.0000000000001412 PMC7282491

[23] Li F, Huang JX, Sun WJ, Zeng JQ, Gan KX, Gong B, et al. A multi-omics analysis of pancreatitis: bridging familial genetics and disease progression. Front Immunol. 2025;16:1707821. 10.3389/fimmu.2025.1707821. 41601670 10.3389/fimmu.2025.1707821 PMC12833420

[24] Cruz-Monserrate Z, Gumpper K, Pita V, Hart PA, Forsmark C, Whitcomb DC, et al. Biomarkers of chronic pancreatitis: a systematic literature review. Pancreatology. 2021;21(2):323–33. 10.1016/j.pan.2021.01.006. 33558189 10.1016/j.pan.2021.01.006 PMC7969447

[25] Saloman JL, Li Y, Stello K, Li W, Li S, Phillips AE, et al. Serum biomarkers of nociceptive and neuropathic pain in chronic pancreatitis. J Pain. 2023;24(12):2199–210. 10.1016/j.jpain.2023.07.006. 37451493 10.1016/j.jpain.2023.07.006 PMC10787046

[26] Xu GY, Winston JH, Shenoy M, Yin H, Pasricha PJ. Enhanced excitability and suppression of A-type K+ current of pancreas-specific afferent neurons in a rat model of chronic pancreatitis. Am J Physiol Gastrointest Liver Physiol. 2006;291(3):G424–31. 10.1152/ajpgi.00560.2005. 16645160 10.1152/ajpgi.00560.2005

[27] Ceyhan GO, Deucker S, Demir IE, Erkan M, Schmelz M, Bergmann F, et al. Neural fractalkine expression is closely linked to pain and pancreatic neuritis in human chronic pancreatitis. Lab Invest. 2009;89(3):347–61. 10.1038/labinvest.2008.170. 19153557 10.1038/labinvest.2008.170

[28] Demir IE, Schorn S, Schremmer-Danninger E, Wang K, Kehl T, Giese NA, et al. Perineural mast cells are specifically enriched in pancreatic neuritis and neuropathic pain in pancreatic cancer and chronic pancreatitis. PLoS ONE. 2013;8(3):e60529. 10.1371/journal.pone.0060529. 23555989 10.1371/journal.pone.0060529 PMC3610867

[29] Dunbar EK, Saloman JL, Phillips AE, Whitcomb DC. Severe pain in chronic pancreatitis patients: considering mental health and associated genetic factors. J Pain Res. 2021;14:773–84. 10.2147/JPR.S274276. 33762844 10.2147/JPR.S274276 PMC7982558

[30] Olesen SS, Krauss T, Demir IE, Wilder-Smith OH, Ceyhan GO, Pasricha PJ, et al. Towards a neurobiological understanding of pain in chronic pancreatitis: mechanisms and implications for treatment. Pain Rep. 2017;2(6):e625. 10.1097/PR9.0000000000000625. 29392239 10.1097/PR9.0000000000000625 PMC5741325

[31] Bouwense SA, de Vries M, Schreuder LT, Olesen SS, Frokjaer JB, Drewes AM, et al. Systematic mechanism-orientated approach to chronic pancreatitis pain. World J Gastroenterol. 2015;21(1):47–59. 10.3748/wjg.v21.i1.47. 25574079 10.3748/wjg.v21.i1.47 PMC4284360

[32] Dunbar E, Greer PJ, Melhem N, Alkaade S, Amann ST, Brand R, et al. Constant-severe pain in chronic pancreatitis is associated with genetic loci for major depression in the NAPS2 cohort. J Gastroenterol. 2020;55(10):1000–9. 10.1007/s00535-020-01703-w. 32681239 10.1007/s00535-020-01703-w PMC9124361

[33] Zhou W, Jin Y, Meng Q, Zhu X, Bai T, Tian Y, et al. A neural circuit for comorbid depressive symptoms in chronic pain. Nat Neurosci. 2019;22(10):1649–58. 10.1038/s41593-019-0468-2. 31451801 10.1038/s41593-019-0468-2

[34] von Hehn CA, Baron R, Woolf CJ. Deconstructing the neuropathic pain phenotype to reveal neural mechanisms. Neuron. 2012;73(4):638–52. 10.1016/j.neuron.2012.02.008. 22365541 10.1016/j.neuron.2012.02.008 PMC3319438

[35] Tsuda M. Microglia-mediated regulation of neuropathic pain: molecular and cellular mechanisms. Biol Pharm Bull. 2019;42(12):1959–68. 10.1248/bpb.b19-00715. 31787711 10.1248/bpb.b19-00715

[36] Boakye PA, Tang SJ, Smith PA. Mediators of neuropathic pain; focus on spinal microglia, CSF-1, BDNF, CCL21, TNF-alpha, Wnt ligands, and Interleukin 1beta. Front Pain Res (Lausanne). 2021;2:698157. 10.3389/fpain.2021.698157. 35295524 10.3389/fpain.2021.698157 PMC8915739

[37] Teckchandani S, Nagana Gowda GA, Raftery D, Curatolo M. Metabolomics in chronic pain research. Eur J Pain. 2021;25(2):313–26. 10.1002/ejp.1677. 33065770 10.1002/ejp.1677 PMC7902309

[38] Faghih M, Phillips AE, Kuhlmann L, Afghani E, Drewes AM, Yadav D, et al. Pancreatic QST differentiates chronic pancreatitis patients into distinct pain phenotypes independent of psychiatric comorbidities. Clin Gastroenterol Hepatol. 2022;20(1):153-61 e2. 10.1016/j.cgh.2020.10.036. 34108130 10.1016/j.cgh.2020.10.036 PMC8629107

[39] Olesen SS, Graversen C, Bouwense SA, van Goor H, Wilder-Smith OH, Drewes AM. Quantitative sensory testing predicts pregabalin efficacy in painful chronic pancreatitis. PLoS ONE. 2013;8(3):e57963. 10.1371/journal.pone.0057963. 23469256 10.1371/journal.pone.0057963 PMC3585877

[40] Teo K, Johnson MH, Drewes AM, Windsor JA. A comprehensive pain assessment tool (COMPAT) for chronic pancreatitis: development, face validation and pilot evaluation. Pancreatology. 2017;17(5):706–19. 10.1016/j.pan.2017.07.004. 28733149 10.1016/j.pan.2017.07.004

[41] Wager TD, Sutherland SP, Lindquist MA, Sluka KA, A2CPS Consortium. Accelerating discovery in pain science: the acute to chronic pain signatures program. Pain. 2025;166(11S):S95–8. 10.1097/j.pain.0000000000003674. 41086337 10.1097/j.pain.0000000000003674 PMC12614242

[42] Diatchenko L, Parisien M, Jahangiri Esfahani S, Mogil JS. Omics approaches to discover pathophysiological pathways contributing to human pain. Pain. 2022;163(Suppl 1):S69–78. 10.1097/j.pain.0000000000002726. 35994593 10.1097/j.pain.0000000000002726 PMC9557800

[43] Bourinet E, Altier C, Hildebrand ME, Trang T, Salter MW, Zamponi GW. Calcium-permeable ion channels in pain signaling. Physiol Rev. 2014;94(1):81–140. 10.1152/physrev.00023.2013. 24382884 10.1152/physrev.00023.2013

[44] Bennett DL, Clark AJ, Huang J, Waxman SG, Dib-Hajj SD. The role of voltage-gated sodium channels in pain signaling. Physiol Rev. 2019;99(2):1079–151. 10.1152/physrev.00052.2017. 30672368 10.1152/physrev.00052.2017

[45] Lainez S, Tsantoulas C, Biel M, McNaughton PA. HCN3 ion channels: roles in sensory neuronal excitability and pain. J Physiol. 2019;597(17):4661–75. 10.1113/JP278211. 31290157 10.1113/JP278211

[46] Schwartz ES, La JH, Scheff NN, Davis BM, Albers KM, Gebhart GF. TRPV1 and TRPA1 antagonists prevent the transition of acute to chronic inflammation and pain in chronic pancreatitis. J Neurosci. 2013;33(13):5603–11. 10.1523/JNEUROSCI.1806-12.2013. 23536075 10.1523/JNEUROSCI.1806-12.2013 PMC3690366

[47] Ceppa E, Cattaruzza F, Lyo V, Amadesi S, Pelayo JC, Poole DP, et al. Transient receptor potential ion channels V4 and A1 contribute to pancreatitis pain in mice. Am J Physiol Gastrointest Liver Physiol. 2010;299(3):G556–71. 10.1152/ajpgi.00433.2009. 20539005 10.1152/ajpgi.00433.2009 PMC2950679

[48] Xu GY, Winston JH, Shenoy M, Yin H, Pendyala S, Pasricha PJ. Transient receptor potential vanilloid 1 mediates hyperalgesia and is up-regulated in rats with chronic pancreatitis. Gastroenterology. 2007;133(4):1282–92. 10.1053/j.gastro.2007.06.015. 17698068 10.1053/j.gastro.2007.06.015

[49] Ma K, Cheng Z, Jiang H, Lin Z, Liu C, Liu X, et al. Expert consensus on ion channel drugs for chronic pain treatment in China. J Pain Res. 2024;17:953–63. 10.2147/JPR.S445171. 38476873 10.2147/JPR.S445171 PMC10929561

[50] Koivisto AP, Belvisi MG, Gaudet R, Szallasi A. Advances in TRP channel drug discovery: from target validation to clinical studies. Nat Rev Drug Discov. 2022;21(1):41–59. 10.1038/s41573-021-00268-4. 34526696 10.1038/s41573-021-00268-4 PMC8442523

[51] Sousa-Valente J, Andreou AP, Urban L, Nagy I. Transient receptor potential ion channels in primary sensory neurons as targets for novel analgesics. Br J Pharmacol. 2014;171(10):2508–27. 10.1111/bph.12532. 24283624 10.1111/bph.12532 PMC4008996

[52] Fogel EL, Easler JJ, Yuan Y, Yadav D, Conwell DL, Vege SS, et al. Safety, tolerability, and dose-limiting toxicity of Lacosamide in patients with painful chronic pancreatitis: protocol for a phase 1 clinical trial to determine safety and identify side effects. JMIR Res Protoc. 2024;13:e50513. 10.2196/50513. 38451604 10.2196/50513 PMC10958339

[53] Bliss TV, Collingridge GL, Kaang BK, Zhuo M. Synaptic plasticity in the anterior cingulate cortex in acute and chronic pain. Nat Rev Neurosci. 2016;17(8):485–96. 10.1038/nrn.2016.68. 27307118 10.1038/nrn.2016.68

[54] Zhang M, Ma Y, Ye X, Zhang N, Pan L, Wang B. TRP (transient receptor potential) ion channel family: structures, biological functions and therapeutic interventions for diseases. Signal Transduct Target Ther. 2023;8(1):261. 10.1038/s41392-023-01464-x. 37402746 10.1038/s41392-023-01464-x PMC10319900

[55] Sousa-Valente J, Brain SD. A historical perspective on the role of sensory nerves in neurogenic inflammation. Semin Immunopathol. 2018;40(3):229–36. 10.1007/s00281-018-0673-1. 29616309 10.1007/s00281-018-0673-1 PMC5960476

[56] Rather MA, Khan A, Wang L, Jahan S, Rehman MU, Makeen HA, et al. TRP channels: role in neurodegenerative diseases and therapeutic targets. Heliyon. 2023;9(6):e16910. 10.1016/j.heliyon.2023.e16910. 37332910 10.1016/j.heliyon.2023.e16910 PMC10272313

[57] Cao B, Xu Q, Shi Y, Zhao R, Li H, Zheng J, et al. Pathology of pain and its implications for therapeutic interventions. Signal Transduct Target Ther. 2024;9(1):155. 10.1038/s41392-024-01845-w. 38851750 10.1038/s41392-024-01845-w PMC11162504

[58] Navratilova E, Porreca F. Substance P and inflammatory pain: getting it wrong and right simultaneously. Neuron. 2019;101(3):353–5. 10.1016/j.neuron.2019.01.034. 30731054 10.1016/j.neuron.2019.01.034

[59] Duitama M, Vargas-Lopez V, Casas Z, Albarracin SL, Sutachan JJ, Torres YP. TRP channels role in pain associated with neurodegenerative diseases. Front Neurosci. 2020;14:782. 10.3389/fnins.2020.00782. 32848557 10.3389/fnins.2020.00782 PMC7417429

[60] Romac JM, Swain SM, Mullappilly N, Bindhani B, Liddle RA. Loss of TRPV4 reduces pancreatic cancer growth and metastasis. JCI Insight. 2025. 10.1172/jci.insight.196280. 41100488 10.1172/jci.insight.196280 PMC12890521

[61] Swain SM, Liddle RA. Mechanosensing Piezo channels in gastrointestinal disorders. J Clin Invest. 2023. 10.1172/JCI171955. 37781915 10.1172/JCI171955 PMC10541197

[62] Zhang WJ, Luo HL, Zhu ZM. The role of P2X4 receptors in chronic pain: a potential pharmacological target. Biomed Pharmacother. 2020;129:110447. 10.1016/j.biopha.2020.110447. 32887026 10.1016/j.biopha.2020.110447

[63] Tozaki-Saitoh H, Takeda H, Inoue K. The role of microglial purinergic receptors in pain signaling. Molecules. 2022. 10.3390/molecules27061919. 35335282 10.3390/molecules27061919 PMC8949888

[64] Burnstock G. Purinergic signalling: pathophysiology and therapeutic potential. Keio J Med. 2013;62(3):63–73. 10.2302/kjm.2013-0003-re. 24067872 10.2302/kjm.2013-0003-re

[65] Molliver DC, Cook SP, Carlsten JA, Wright DE, McCleskey EW. ATP and UTP excite sensory neurons and induce CREB phosphorylation through the metabotropic receptor, P2Y2. Eur J Neurosci. 2002;16(10):1850–60. 10.1046/j.1460-9568.2002.02253.x. 12453048 10.1046/j.1460-9568.2002.02253.x

[66] Zhu H, Yu Y, Zheng L, Wang L, Li C, Yu J, et al. Chronic inflammatory pain upregulates expression of P2Y2 receptor in small-diameter sensory neurons. Metab Brain Dis. 2015;30(6):1349–58. 10.1007/s11011-015-9695-8. 26062804 10.1007/s11011-015-9695-8

[67] Deval E, Lingueglia E. Acid-sensing ion channels and nociception in the peripheral and central nervous systems. Neuropharmacology. 2015;94:49–57. 10.1016/j.neuropharm.2015.02.009. 25724084 10.1016/j.neuropharm.2015.02.009

[68] Holzer P. Acid-sensing ion channels in gastrointestinal function. Neuropharmacology. 2015;94:72–9. 10.1016/j.neuropharm.2014.12.009. 25582294 10.1016/j.neuropharm.2014.12.009 PMC4458375

[69] Zamponi GW, Lewis RJ, Todorovic SM, Arneric SP, Snutch TP. Role of voltage-gated calcium channels in ascending pain pathways. Brain Res Rev. 2009;60(1):84–9. 10.1016/j.brainresrev.2008.12.021. 19162069 10.1016/j.brainresrev.2008.12.021 PMC2692704

[70] Qi FH, Zhou YL, Xu GY. Targeting voltage-gated sodium channels for treatment for chronic visceral pain. World J Gastroenterol. 2011;17(19):2357–64. 10.3748/wjg.v17.i19.2357. 21633634 10.3748/wjg.v17.i19.2357 PMC3103787

[71] Erickson A, Deiteren A, Harrington AM, Garcia-Caraballo S, Castro J, Caldwell A, et al. Voltage-gated sodium channels: (Na(V) )igating the field to determine their contribution to visceral nociception. J Physiol. 2018;596(5):785–807. 10.1113/JP273461. 29318638 10.1113/JP273461 PMC5830430

[72] Keam SJ. Suzetrigine: first approval. Drugs. 2025;85(6):845–51. 10.1007/s40265-025-02178-w. 40323340 10.1007/s40265-025-02178-w

[73] Gordon EA, Tyagi S, Dib-Hajj SD, Bennett DL. Selective targeting of voltage-gated sodium channels to achieve analgesia: current status and future directions. Pain. 2025;166(11S):S42–6. 10.1097/j.pain.0000000000003639. 41086326 10.1097/j.pain.0000000000003639

[74] Terada Y, Fujimura M, Nishimura S, Tsubota M, Sekiguchi F, Kawabata A. Roles of Cav3.2 and TRPA1 channels targeted by hydrogen sulfide in pancreatic nociceptive processing in mice with or without acute pancreatitis. J Neurosci Res. 2015;93(2):361–9. 10.1002/jnr.23490. 25267397 10.1002/jnr.23490

[75] Sekiguchi F, Tsubota M, Kawabata A. Involvement of voltage-gated calcium channels in inflammation and inflammatory pain. Biol Pharm Bull. 2018;41(8):1127–34. 10.1248/bpb.b18-00054. 30068860 10.1248/bpb.b18-00054

[76] Ricardo Carvalho VP, da Figueira Silva J, Buzelin MA, da Antonio Silva Junior C, Dos Carvalho Santos D, Montijo Diniz D, et al. Calcium channels blockers toxins attenuate abdominal hyperalgesia and inflammatory response associated with the cerulein-induced acute pancreatitis in rats. Eur J Pharmacol. 2021;891:173672. 10.1016/j.ejphar.2020.173672. 33190801 10.1016/j.ejphar.2020.173672

[77] Cao YQ. Voltage-gated calcium channels and pain. Pain. 2006;126(1–3):5–9. 10.1016/j.pain.2006.10.019. 17084979 10.1016/j.pain.2006.10.019

[78] Weniger M, Reinelt L, Neumann J, Holdt L, Ilmer M, Renz B, et al. The analgesic effect of the mitochondria-targeted antioxidant SkQ1 in pancreatic inflammation. Oxid Med Cell Longev. 2016;2016:4650489. 10.1155/2016/4650489. 27274778 10.1155/2016/4650489 PMC4870369

[79] Robles L, Vaziri ND, Ichii H. Role of oxidative stress in the pathogenesis of pancreatitis: effect of antioxidant therapy. Pancreat Disord Ther. 2013;3(1):112. 10.4172/2165-7092.1000112. 24808987 10.4172/2165-7092.1000112 PMC4009983

[80] Biczo G, Vegh ET, Shalbueva N, Mareninova OA, Elperin J, Lotshaw E, et al. Mitochondrial dysfunction, through impaired autophagy, leads to endoplasmic reticulum stress, deregulated lipid metabolism, and pancreatitis in animal models. Gastroenterology. 2018;154(3):689–703. 10.1053/j.gastro.2017.10.012. 29074451 10.1053/j.gastro.2017.10.012 PMC6369139

[81] Sui BD, Xu TQ, Liu JW, Wei W, Zheng CX, Guo BL, et al. Understanding the role of mitochondria in the pathogenesis of chronic pain. Postgrad Med J. 2013;89(1058):709–14. 10.1136/postgradmedj-2012-131068. 24151337 10.1136/postgradmedj-2012-131068

[82] Kang R, Lotze MT, Zeh HJ, Billiar TR, Tang D. Cell death and DAMPs in acute pancreatitis. Mol Med. 2014;20(1):466–77. 10.2119/molmed.2014.00117. 25105302 10.2119/molmed.2014.00117 PMC4277549

[83] Chen H, Wang Y, Zippi M, Fiorino S, Hong W. Oxidative stress, DAMPs, and immune cells in acute pancreatitis: molecular mechanisms and therapeutic prospects. Front Immunol. 2025;16:1608618. 10.3389/fimmu.2025.1608618. 40909291 10.3389/fimmu.2025.1608618 PMC12405227

[84] Wang J, Song Y, Chen Z, Leng SX. Connection between systemic inflammation and neuroinflammation underlies neuroprotective mechanism of several phytochemicals in neurodegenerative diseases. Oxid Med Cell Longev. 2018;2018:1972714. 10.1155/2018/1972714. 30402203 10.1155/2018/1972714 PMC6196798

[85] Gamper N, Ooi L. Redox and nitric oxide-mediated regulation of sensory neuron ion channel function. Antioxid Redox Signal. 2015;22(6):486–504. 10.1089/ars.2014.5884. 24735331 10.1089/ars.2014.5884 PMC4323017

[86] Bhardwaj P, Yadav RK. Chronic pancreatitis: role of oxidative stress and antioxidants. Free Radic Res. 2013;47(11):941–9. 10.3109/10715762.2013.804624. 23668832 10.3109/10715762.2013.804624

[87] Yousuf MS, Maguire AD, Simmen T, Kerr BJ. Endoplasmic reticulum-mitochondria interplay in chronic pain: the calcium connection. Mol Pain. 2020;16:1744806920946889. 10.1177/1744806920946889. 32787562 10.1177/1744806920946889 PMC7427143

[88] Bahar E, Kim H, Yoon H. ER stress-mediated signaling: action potential and Ca(2+) as key players. Int J Mol Sci. 2016. 10.3390/ijms17091558. 27649160 10.3390/ijms17091558 PMC5037829

[89] Verma M, Lizama BN, Chu CT. Excitotoxicity, calcium and mitochondria: a triad in synaptic neurodegeneration. Transl Neurodegener. 2022;11(1):3. 10.1186/s40035-021-00278-7. 35078537 10.1186/s40035-021-00278-7 PMC8788129

[90] Wang W, Kong M, Dou Y, Xue S, Liu Y, Zhang Y, et al. Selective expression of a SNARE-cleaving protease in peripheral sensory neurons attenuates pain-related gene transcription and neuropeptide release. Int J Mol Sci. 2021. 10.3390/ijms22168826. 34445536 10.3390/ijms22168826 PMC8396265

[91] Kinoshita PF, Leite JA, Orellana AM, Vasconcelos AR, Quintas LE, Kawamoto EM, et al. The influence of Na(+), K(+)-ATPase on glutamate signaling in neurodegenerative diseases and senescence. Front Physiol. 2016;7:195. 10.3389/fphys.2016.00195. 27313535 10.3389/fphys.2016.00195 PMC4890531

[92] Wu XF, Liu WT, Liu YP, Huang ZJ, Zhang YK, Song XJ. Reopening of ATP-sensitive potassium channels reduces neuropathic pain and regulates astroglial gap junctions in the rat spinal cord. Pain. 2011;152(11):2605–15. 10.1016/j.pain.2011.08.003. 21907492 10.1016/j.pain.2011.08.003

[93] LaRusch J, Whitcomb DC. Genetics of pancreatitis. Curr Opin Gastroenterol. 2011;27(5):467–74. 10.1097/MOG.0b013e328349e2f8. 21844754 10.1097/MOG.0b013e328349e2f8 PMC3704192

[94] Geisz A, Sahin-Toth M. A preclinical model of chronic pancreatitis driven by trypsinogen autoactivation. Nat Commun. 2018;9(1):5033. 10.1038/s41467-018-07347-y. 30487519 10.1038/s41467-018-07347-y PMC6261995

[95] Rosendahl J, Kirsten H, Hegyi E, Kovacs P, Weiss FU, Laumen H, et al. Genome-wide association study identifies inversion in the CTRB1-CTRB2 locus to modify risk for alcoholic and non-alcoholic chronic pancreatitis. Gut. 2018;67(10):1855–63. 10.1136/gutjnl-2017-314454. 28754779 10.1136/gutjnl-2017-314454 PMC6145291

[96] Sahin-Toth M. Genetic risk in chronic pancreatitis: the misfolding-dependent pathway. Curr Opin Gastroenterol. 2017;33(5):390–5. 10.1097/MOG.0000000000000380. 28650851 10.1097/MOG.0000000000000380 PMC5549634

[97] Sahin-Toth M. Channelopathy of the pancreas causes chronic pancreatitis. Gastroenterology. 2020;158(6):1538–40. 10.1053/j.gastro.2020.03.027. 32205170 10.1053/j.gastro.2020.03.027 PMC7751598

[98] Masamune A, Kotani H, Sorgel FL, Chen JM, Hamada S, Sakaguchi R, et al. Variants that affect function of calcium channel TRPV6 are associated with early-onset chronic pancreatitis. Gastroenterology. 2020;158(6):1626-41 e8. 10.1053/j.gastro.2020.01.005. 31930989 10.1053/j.gastro.2020.01.005

[99] Butnariu LI, Tarca E, Cojocaru E, Rusu C, Moisa SM, Leon Constantin MM, et al. Genetic modifying factors of cystic fibrosis phenotype: a challenge for modern medicine. J Clin Med. 2021. 10.3390/jcm10245821. 34945117 10.3390/jcm10245821 PMC8707808

[100] Reimann F, Cox JJ, Belfer I, Diatchenko L, Zaykin DV, McHale DP, et al. Pain perception is altered by a nucleotide polymorphism in SCN9A. Proc Natl Acad Sci U S A. 2010;107(11):5148–53. 10.1073/pnas.0913181107. 20212137 10.1073/pnas.0913181107 PMC2841869

[101] Kuhlmann L, Olesen SS, Drewes AM. Assessment of visceral pain with special reference to chronic pancreatitis. Front Pain Res. 2022;3:1067103. 10.3389/fpain.2022.1067103. 10.3389/fpain.2022.1067103 PMC9807876 36606031

[102] Tuck NL, Teo K, Kuhlmann L, Olesen SS, Johnson M, Bean DJ, et al. Pain patterns in chronic pancreatitis and chronic primary pain. Pancreatology. 2022;22(5):572–82. 10.1016/j.pan.2022.04.016. 35562269 10.1016/j.pan.2022.04.016

[103] Fitzcharles MA, Cohen SP, Clauw DJ, Littlejohn G, Usui C, Hauser W. Nociplastic pain: towards an understanding of prevalent pain conditions. Lancet. 2021;397(10289):2098–110. 10.1016/S0140-6736(21)00392-5. 34062144 10.1016/S0140-6736(21)00392-5

[104] Rahib L, Salerno W, Abu-El-Haija M, Conwell DL, Freeman AJ, Hart PA, et al. Development of a core outcome set for recurrent acute and chronic pancreatitis: results of a Delphi poll. Pancreatology. 2024;24(8):1237–43. 10.1016/j.pan.2024.11.013. 39609172 10.1016/j.pan.2024.11.013 PMC11648530

[105] Beas R, Riva-Moscoso A, Ribaudo I, Chambergo-Michilot D, Norwood DA, Karkash A, et al. Prevalence of depression among patients with chronic pancreatitis: a systematic review and meta-analysis. Clin Res Hepatol Gastroenterol. 2023;47(5):102115. 10.1016/j.clinre.2023.102115. 36977457 10.1016/j.clinre.2023.102115

[106] Anderson MA, Akshintala V, Albers KM, Amann ST, Belfer I, Brand R, et al. Mechanism, assessment and management of pain in chronic pancreatitis: recommendations of a multidisciplinary study group. Pancreatology. 2016;16(1):83–94. 10.1016/j.pan.2015.10.015. 26620965 10.1016/j.pan.2015.10.015 PMC4761301

[107] Yadav D, Askew RL, Palermo T, Li L, Andersen DK, Chen M, et al. Association of chronic pancreatitis pain features with physical, mental, and social health. Clin Gastroenterol Hepatol. 2023;21(7):1781-91 e4. 10.1016/j.cgh.2022.09.026. 36191836 10.1016/j.cgh.2022.09.026 PMC10065964

[108] Phillips AE, Bick BL, Faghih M, Yadav D, Drewes AM, Singh VK, et al. Pain sensitivity and psychiatric comorbidities in chronic pancreatitis patients with and without pain: past experience matters. Gastro Hep Advances. 2022;1(5):796–802. 10.1016/j.gastha.2022.04.013. 39131846 10.1016/j.gastha.2022.04.013 PMC11307602

[109] Apkarian AV, Baliki MN, Geha PY. Towards a theory of chronic pain. Prog Neurobiol. 2009;87(2):81–97. 10.1016/j.pneurobio.2008.09.018. 18952143 10.1016/j.pneurobio.2008.09.018 PMC2650821

[110] Giossi R, Carrara F, Padroni M, Bilancio MC, Mazzari M, Enisci S, et al. Systematic review and meta-analysis seem to indicate that cannabinoids for chronic primary pain treatment have limited benefit. Pain Ther. 2022;11(4):1341–58. 10.1007/s40122-022-00434-5. 36129666 10.1007/s40122-022-00434-5 PMC9633894

[111] Neblett R, Sanabria-Mazo JP, Luciano JV, Mircic M, Colovic P, Bojanic M, et al. Is the central sensitization inventory (CSI) associated with quantitative sensory testing (QST)? A systematic review and meta-analysis. Neurosci Biobehav Rev. 2024;161:105612. 10.1016/j.neubiorev.2024.105612. 38604015 10.1016/j.neubiorev.2024.105612

[112] Yunus MB. Fibromyalgia and overlapping disorders: the unifying concept of central sensitivity syndromes. Semin Arthritis Rheum. 2007;36(6):339–56. 10.1016/j.semarthrit.2006.12.009. 17350675 10.1016/j.semarthrit.2006.12.009

[113] Gaskell H, Moore RA, Derry S, Stannard C. Oxycodone for pain in fibromyalgia in adults. Cochrane Database Syst Rev. 2016;9(9):CD012329. 10.1002/14651858.CD012329. 27582266 10.1002/14651858.CD012329 PMC6457853

[114] Fernandez-Feijoo F, Samartin-Veiga N, Carrillo-de-la-Pena MT. Quality of life in patients with fibromyalgia: contributions of disease symptoms, lifestyle and multi-medication. Front Psychol. 2022;13:924405. 10.3389/fpsyg.2022.924405. 36262444 10.3389/fpsyg.2022.924405 PMC9574370

[115] Montagne JM, Jaffee EM, Fertig EJ. Multiomics empowers predictive pancreatic cancer immunotherapy. J Immunol. 2023;210(7):859–68. 10.4049/jimmunol.2200660. 36947820 10.4049/jimmunol.2200660 PMC10236355

[116] Mackey S, Aghaeepour N, Gaudilliere B, Kao MC, Kaptan M, Lannon E, et al. Innovations in acute and chronic pain biomarkers: enhancing diagnosis and personalized therapy. Reg Anesth Pain Med. 2025;50(2):110–20. 10.1136/rapm-2024-106030. 39909549 10.1136/rapm-2024-106030 PMC11877092

[117] Nag DS, Swain BP, Anand R, Barman TK, Vatsala. Pain management in chronic pancreatitis. World J Clin Cases. 2024;12(12):2016–22. 10.12998/wjcc.v12.i12.2016. 38680261 10.12998/wjcc.v12.i12.2016 PMC11045512

[118] Swaminathan G, Saito T, Husain SZ. Exploiting open source omics data to advance pancreas research. J Pancreatology. 2024;7(1):21–7. 10.1097/JP9.0000000000000173. 10.1097/JP9.0000000000000173 PMC10959533 38524857

[119] Arjmand B, Hamidpour SK, Tayanloo-Beik A, Goodarzi P, Aghayan HR, Adibi H, et al. Machine learning: a new prospect in multi-omics data analysis of cancer. Front Genet. 2022;13:824451. 10.3389/fgene.2022.824451. 35154283 10.3389/fgene.2022.824451 PMC8829119

[120] Acharya D, Mukhopadhyay A. A comprehensive review of machine learning techniques for multi-omics data integration: challenges and applications in precision oncology. Brief Funct Genomics. 2024;23(5):549–60. 10.1093/bfgp/elae013. 38600757 10.1093/bfgp/elae013

[121] Valous NA, Popp F, Zornig I, Jager D, Charoentong P. Graph machine learning for integrated multi-omics analysis. Br J Cancer. 2024;131(2):205–11. 10.1038/s41416-024-02706-7. 38729996 10.1038/s41416-024-02706-7 PMC11263675

[122] Athieniti E, Spyrou GM. A guide to multi-omics data collection and integration for translational medicine. Comput Struct Biotechnol J. 2023;21:134–49. 10.1016/j.csbj.2022.11.050. 36544480 10.1016/j.csbj.2022.11.050 PMC9747357

[123] Yadav D, Park WG, Fogel EL, Li L, Chari ST, Feng Z, et al. PROspective Evaluation of Chronic Pancreatitis for EpidEmiologic and Translational StuDies: rationale and study design for PROCEED from the Consortium for the Study of Chronic Pancreatitis, Diabetes, and Pancreatic Cancer. Pancreas. 2018;47(10):1229–38. 10.1097/MPA.0000000000001170. 30325862 10.1097/MPA.0000000000001170 PMC6619499

[124] Bellin MD, Lowe M, Zimmerman MB, Wilschanski M, Werlin S, Troendle DM, et al. Diabetes mellitus in children with acute recurrent and chronic pancreatitis: data from the INternational Study Group of Pediatric Pancreatitis: In search for a CuRE cohort. J Pediatr Gastroenterol Nutr. 2019;69(5):599–606. 10.1097/MPG.0000000000002482. 31651815 10.1097/MPG.0000000000002482 PMC6834233

[125] Hart PA, Papachristou GI, Park WG, Dyer AM, Chinchilli VM, Afghani E, et al. Rationale and design for the diabetes related to acute pancreatitis and its mechanisms study: a prospective cohort study from the type 1 diabetes in acute pancreatitis consortium. Pancreas. 2022;51(6):568–74. 10.1097/MPA.0000000000002079. 36206460 10.1097/MPA.0000000000002079 PMC9555871

[126] Edwards RR, Schreiber KL, Dworkin RH, Turk DC, Baron R, Freeman R, et al. Optimizing and accelerating the development of precision pain treatments for chronic pain: IMMPACT review and recommendations. J Pain. 2023;24(2):204–25. 10.1016/j.jpain.2022.08.010. 36198371 10.1016/j.jpain.2022.08.010 PMC10868532

[127] Behrens LMP, Fernandes GDS, Goncalves GF, Nunes FVM, Weimer RD, Moreira JCF, et al. Limitations and opportunities in multi-omics integration for neurodevelopmental, neurodegenerative and psychiatric disorders: a systematic review. Neuroscience. 2026. 10.1016/j.neuroscience.2026.01.019. 41638346 10.1016/j.neuroscience.2026.01.019

[128] Misra BB, Langefeld C, Olivier M, Cox LA. Integrated omics: tools, advances and future approaches. J Mol Endocrinol. 2019;62(1):R21–45. 10.1530/JME-18-0055. 30006342 10.1530/JME-18-0055

[129] Ramos-Lopez O, Martinez JA, Milagro FI. Holistic integration of Omics tools for precision nutrition in health and disease. Nutrients. 2022. 10.3390/nu14194074. 36235725 10.3390/nu14194074 PMC9572439

[130] Gottardi Zamperla M, Barbi V, Negri S, Atlante S, Gaetano C. Omics medicine: what the clinicians should know. Eur J Intern Med. 2026. 10.1016/j.ejim.2026.106759. 41680041 10.1016/j.ejim.2026.106759

[131] Cominetti O, Dayon L. Unravelling disease complexity: integrative analysis of multi-omic data in clinical research. Expert Rev Proteomics. 2025;22(4):149–62. 10.1080/14789450.2025.2491357. 40207843 10.1080/14789450.2025.2491357

[132] Franks PW, Cefalu WT, Dennis J, Florez JC, Mathieu C, Morton RW, et al. Precision medicine for cardiometabolic disease: a framework for clinical translation. Lancet Diabetes Endocrinol. 2023;11(11):822–35. 10.1016/S2213-8587(23)00165-1. 37804856 10.1016/S2213-8587(23)00165-1

[133] Eldabe S, Obara I, Panwar C, Caraway D. Biomarkers for chronic pain: significance and summary of recent advances. Pain Res Manag. 2022;2022:1940906. 10.1155/2022/1940906. 36385904 10.1155/2022/1940906 PMC9663208

[134] de Vries M, Wilder-Smith OH, Jongsma ML, van den Broeke EN, Arns M, van Goor H, et al. Altered resting state EEG in chronic pancreatitis patients: toward a marker for chronic pain. J Pain Res. 2013;6:815–24. 10.2147/JPR.S50919. 24379694 10.2147/JPR.S50919 PMC3843642

[135] Muthulingam JA, Hansen TM, Drewes AM, Olesen SS, Frokjaer JB. Disrupted functional connectivity of Default Mode and Salience Networks in chronic pancreatitis patients. Clin Neurophysiol. 2020;131(5):1021–9. 10.1016/j.clinph.2020.01.016. 32197125 10.1016/j.clinph.2020.01.016

